# A systematic computational analysis of pharmacological options in neuroinflammatory-induced autism spectrum disorder in children: A potential for drug repositioning

**DOI:** 10.14440/jbm.0219

**Published:** 2025-10-23

**Authors:** Manel Ismail, Soukaïna Aananou, Clovis Foguem, Kokou M. Guinhouya, Gloria T. Dossou, Fabio Boudis, Kissaou Tchedre, Christian Vilhelm, Benjamin C. Guinhouya, Djamel Zitoun

**Affiliations:** 1 Department of Health Engineering and Management, Faculty of Health and Sport Sciences, The University of Lille, Lille, Nord 59000, France; 2 Univ. Lille, ULR 2694 METRICS, The University of Lille, Lille, Nord 59000, France; 3 Department of Surgery, Gastroenterology, and Emergency, Auban–Moët Hospital, Epernay, Marne 51200, France; 4 Department of Neurology, Sylvanus Olympio University Hospital, Lomé 99345, Togo; 5 Univ. Lille, Lille University Management Lab (LUMEN) ULR 4999, The University of Lille, Lille, Nord 59000, France; 6 Department of Medical Informatics, Lille University Hospital, Lille, Nord 59000, France; 7 Nanoscope Technologies, LLC, Bedford, Texas 76022, United States of America; 8 Cancer Heterogeneity, Plasticity, and Resistance to Therapies (CANTHER), National Centre for Scientific Research, National Institute of Health and Medical Research, Lille University Hospital, The University of Lille, Lille, Nord 59000, France

**Keywords:** Brain, Bioinformatics, Data science, Inflammation, Pharmacology, Knowledge graph, Youth

## Abstract

**Background:**

Autism spectrum disorder (ASD) is a neurodevelopmental condition characterized by deficits in social communication and the presence of restricted or repetitive behaviors. Although its underlying pathophysiological mechanisms remain unclear, growing evidence indicates that neuroinflammation plays a significant role, especially in children.

**Objective:**

This study aims to explore neuroinflammatory pathways in children aged 12 and under, with a focus on potential therapeutic opportunities through drug repositioning.

**Methods:**

We conducted a systematic computational analysis using data from 27 studies and bioinformatics resources such as DrugBank and PubChem, identifying over 8,000 potential drug candidates from the initial 29 treatments retrieved from the literature.

**Results:**

Key compounds such as cannabidiol, fluoxetine, and risperidone were highlighted for their broad therapeutic potential. In addition, emerging treatments, including cell-based therapies and dietary interventions, were explored.

**Conclusion:**

Our findings support drug repositioning as an effective strategy for developing new ASD treatments during critical developmental periods, emphasizing the need for further research to validate these pathways and the efficacy of innovative therapies.

## 1. Introduction

Autism spectrum disorder (ASD) is a neurodevelopmental condition, identifiable in children through challenges in social communication and restricted, repetitive patterns of behavior and interests.^1^ Due to a spectrum of manifestations and severity levels, ASD is acknowledged as a heterogeneous phenotype, posing substantial issues for diagnosis and treatment.^2^ Over recent decades, the prevalence of ASD has risen markedly. In the United States alone, rates have escalated from 1 in 150 children in 2000 to 1 in 36 in 2023, reflecting in part changes in diagnostic criteria and reporting practices.^3^

ASD frequently co-occurs uniquely or in combination with other neurodevelopmental disorders such as attention deficit/hyperactivity disorder (ADHD), intellectual disabilities, or specific learning disorders, complicating its clinical presentation and management.^4^ In addition, children with ASD often exhibit various comorbidities such as epilepsy, sleep disturbances, anxiety, depression, bipolar disorder, sensory processing disorders, and schizophrenia. The complexity of ASD is further highlighted by findings that autistic children are 67% more likely to develop inflammatory bowel diseases, such as Crohn’s disease or ulcerative colitis, than their peers.^5^ These overlapping conditions, particularly in cases of ADHD co-occurrence with Crohn’s disease and psoriasis, where the prevalence is higher among girls, underscore the multifaceted nature of ASD.^4^ The presence of these comorbidities and associated conditions not only complicates the diagnosis and treatment of ASD but also emphasizes the need for a multifaceted therapeutic approach. Understanding the broader neurodevelopmental and immunological context of ASD is critical for developing targeted treatments that address not just the core symptoms of ASD but also the complex interplay of co-occurring disorders and inflammatory pathways. This complexity is compounded by the fact that, despite extensive research efforts, the underlying causes of ASD remain elusive. There is still no consensus on the exact interplay of genetic, environmental, and biological factors.^6^ Important environmental exposures, such as xenobiotics during prenatal life, heavy metals, and environmental toxicants during critical developmental periods, have been associated with ASD^7^. These exposures are thought to interfere with metabolic and biochemical pathways, potentially leading to oxidative stress, mitochondrial anomalies, and immune dysregulation.^8^

In recent years, the focus has shifted toward understanding the role of neuroinflammatory pathways in ASD. Studies have explored how activating the brain’s immune system may alter neurological development and function. Evidence of immunological alterations, including atypical microglial activity and elevated cytokine levels, suggests that these disturbances may play a critical role in disrupting the neural circuits integral to ASD.^9^-^12^

In light of these findings, the potential for therapeutic interventions targeting neuroinflammatory pathways becomes crucial, especially since current treatments for ASD, particularly in pediatric populations, are largely aimed at symptom management to improve daily functioning and quality of life.^13^ However, despite the adoption of holistic frameworks, ASD is still predominantly treated as a mental disorder, with a strong emphasis on cognitive-behavioral therapies. While these therapies, such as applied behavior analysis, have demonstrated some effectiveness, their success is often contingent on intensive and prolonged intervention, sometimes requiring up to 40 h/week, which can be difficult to sustain and may yield only modest improvements.^14^,^15^ This reality underscores the urgent need for additional therapeutic strategies, including pharmacological interventions, which are increasingly recognized as vital for managing core and co-occurring symptoms like irritability and anxiety.^14^,^16^,^17^

One promising strategy in ASD treatment is drug repositioning. This approach leverages existing knowledge of the safety and pharmacokinetic profiles of approved drugs, reducing both the cost and time required for development, while facilitating quicker access to effective treatments.^10^,^18^ Given the complexity of ASD, repositioning existing drugs to target well-understood biological pathways, including those involved in neuroinflammation, holds significant promise.^11^,^19^ For example, drugs initially developed for other conditions, such as anti-inflammatory or immunomodulatory agents, may offer therapeutic benefits for targeting neuroinflammatory pathways implicated in ASD.^20^

To explore this potential, this study aims to develop a systematic computational approach, investigating the role of neuroinflammation in ASD and the potential for drug repositioning in children with ASD. The focus is on three key elements: therapeutic categories, protein targets, and biological pathways.

## 2. Methodology

### 2.1. Study design and data sources

**Figure 1:**
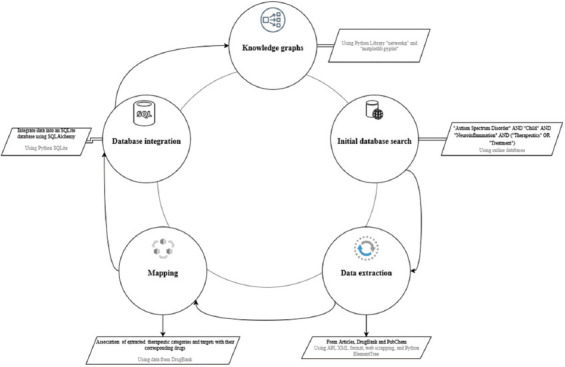
Overview of the methodological steps

This study involved a comprehensive two-tiered selection process (Figure 1) of original research studies and systematic reviews, focusing on neuroinflammation in ASD among children aged ≤12 years. This approach was taken to reduce the clinical and biological heterogeneity of the analyzed data. ASD presents considerable variability depending on age, developmental stage, and the co-occurrence of comorbidities. Expanding the age range would have introduced an additional layer of complexity, making the systematic computational analysis less consistent. Thus, we opted to focus on the pre-adolescent period to better circumscribe our study population. By targeting a relatively homogeneous age group, we were able to structure the data around therapeutic categories, proteomic targets, and biological pathways that are specific to this developmental phase. Finally, this choice enabled the formulation of therapeutic hypotheses that were more relevant for early intervention strategies, which are currently a priority in the management of ASD.

**Figure 2:**
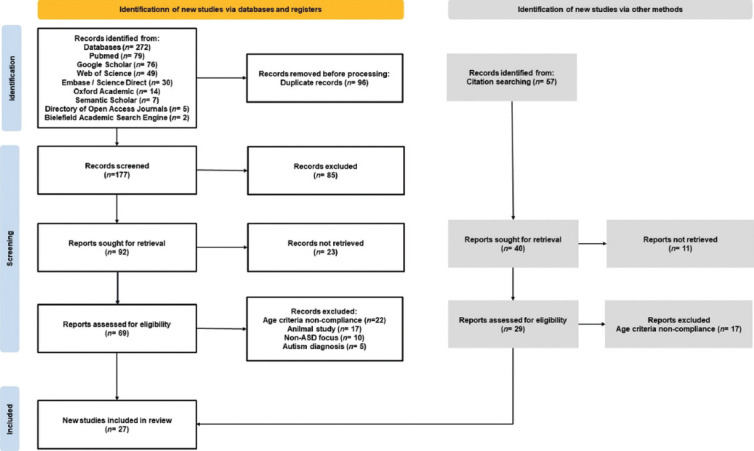
Flowchart for the selection of studies Abbreviation: ASD: Autism spectrum disorder.

The selection of studies was performed according to the Preferred Reporting Items for Systematic Reviews and Meta-Analyses guidelines to ensure rigorous methodology (Figure 2).

#### 2.1.1. Primary data sources

Initially, we identified relevant original research studies using predefined inclusion criteria (i.e., children aged ≤12 years and studies published in English between 2008 and 2023) by targeting various study designs such as case studies, observational reports, and randomized controlled trials. Non-human experimental studies, research involving adolescents or adults with ASD, and studies focusing solely on behavioral therapies without addressing neuroinflammation or biomedical treatments were excluded.

The search for relevant studies employed the following search equation: “Autism Spectrum Disorder” AND “Child” AND “Neuroinflammation” AND (“Therapeutics” OR “Treatment”). Supplementary methods, including citation searching, were also used. Duplicate records were removed, and the remaining records underwent title and abstract screening. Full-text assessments were independently conducted by two authors to determine eligibility, with discrepancies resolved through discussion.

#### 2.1.2. Data mapping process

To enhance the clarity and comprehensiveness of our data analysis, we developed a methodological approach involving a systematic data mapping process. This process included two main steps:

(i) Data extraction from articles: We began by extracting the treatments described in the selected articles, focusing on their specific components (e.g., drugs or nutritional supplements). For each component, we retrieved therapeutic categories, proteomic targets, and biological pathways using data from DrugBank, PubChem, and the Small Molecule Pathway Database (SMPDB), respectively.
(ii) Cross-referencing with external data sources: In parallel, we identified additional therapeutic categories, proteomic targets, and biological pathways that corresponded to the retrieved components. The additional data points helped identify new drugs linked to the same therapeutic categories, proteomic targets, or pathways, which were not initially part of the treatment components. These drugs were considered potential candidates for therapeutic repositioning. We then integrated this information by mapping drug categories, targets, and pathways to their respective drugs through XML scans and cross-referencing with DrugBank and SMPDB.

#### 2.1.3. Database management

The extracted and processed data were integrated into an SQLite database (version 3.38.4) managed through SQLAlchemy (version 2.0.30), an object-relational mapping library for Python (version 3.11.0). This database was designed not just as a data repository but as a comprehensive knowledge base, facilitating the analysis of complex relationships between various treatments and their biological pathways. Custom scripts ensured efficient data insertion and updates.

#### 2.1.4. Secondary data sources

The secondary data sources consisted of systematic reviews, meta-analyses, and other secondary sources used to elucidate the biological mechanisms underlying neuroinflammation in ASD. Specifically, 13 secondary articles were reviewed to identify and extract relevant biological pathways. From these articles, we predefined six general biological pathways: “inflammation,” “immune system,” “oxidative stress,” “neurotransmitter regulation,” “gene expression regulation,” and the “gut–brain axis” based on the following criteria:

(i) Relevance to ASD: Pathways were considered relevant if they were directly linked to key aspects of ASD pathology or symptomatology, such as behavioral regulation, neurodevelopment, or immune responses
(ii) Frequency of mention: We prioritized pathways that were mentioned in multiple articles, suggesting a consensus or recurring focus in the literature
(iii) Impact on neuroinflammation: Pathways were selected based on their demonstrated or hypothesized role in modulating neuroinflammatory processes, particularly those known to affect neural circuits involved in ASD.

While these articles provided important context and background, they were not included in the final core study count. Instead, they served to inform our understanding of the broader biological mechanisms and guided the interpretation of findings from primary research.

### 2.2. Data collection and processing

Study metrics, protocols, and treatments were extracted from the selected articles. Two co-authors independently reviewed the full texts to ensure data accuracy and relevance. The names of the drugs or nutritional supplements used in the treatments were standardized using the DrugBank database.

#### 2.2.1. Pathway classification

Following the identification of relevant compounds and building on the foundational knowledge provided by the 13 key articles, as well as additional information from scientific literature and the SMPDB, keywords and actions associated with each compound were extracted to align them with the predefined pathways.

This categorization process was conducted by two independent authors who identified specific terms describing each compound’s action or mechanism. For treatments and compounds with data available in the SMPDB, relevant biological mechanisms were identified. When specific pathway data for a compound were not available in SMPDB, a literature search was conducted to find pertinent information using search terms like “treatment_name AND biological pathway” or “treatment_name AND mechanism of action.” Each compound’s alignment to a pathway was supported by bibliographic sources or SMPDB data.

#### 2.2.2. Data retrieval

Based on the identification of relevant compounds, Python scripts were used to parse and retrieve detailed information from DrugBank academic resources and data from PubChem. Specifically, the “requests” library (version 2.31.0) was employed to interact with the PubChem application programming interface, fetching target data for specific PubChem Compound IDs to ensure clinical relevance. The Python ElementTree XML parser was utilized to extract drug information from DrugBank XML files, focusing on drug categories. Predefined drug IDs were referenced to identify and extract relevant categories accurately.

#### 2.2.3. Data analysis and visualization

To enhance data analysis and visualization, knowledge graphs were constructed using the networkx library (version 3.3) and visualized using matplotlib.pyplot (version 3.8.3). To explore potential drug repositioning for ASD, three types of knowledge graphs were created for each treatment: therapeutic categories, biological pathways, and proteomic targets.

The therapeutic categories graph was displayed according to its “components” (breaks down the treatment into main components); “categories” (link components to therapeutic categories); “associated drugs” (i.e., additional drugs in each category for repositioning); and “reported effects” (i.e., summary of therapeutic effects observed). The biological pathways graph was composed with its “components” (details about main treatment components); “biological pathways keywords” (link components to keywords describing their biological actions); “biological pathways box” (connection of predefined pathways); “SMPDB pathways and drugs” (i.e., additional drugs associated with the same pathways); and “reported effects.” Finally, the proteomic targets graph included its “components” (outline of main treatment components); “targets” (links of components to proteomic targets); “associated drugs” (depicting additional drugs targeting the same proteomic entities); and “reported effects.”

However, a comprehensive dashboard using Python and JavaScript (version 8.0 for Chrome 140) was developed to visualize the full spectrum of outcomes. The interactive dashboard is available at https://autism-research.univ-lille.fr/index.html, while certain advanced features can be provided on request. This interactive interface should allow users to progressively select and explore the full range of information of interest, enabling them to examine specific pathways, proteomic targets, and therapeutic categories. The dashboard could generate a list of drugs associated with these elements, providing a more detailed exploration and a user-friendly navigation. Additional development was conducted on common proteomic targets to assist in identifying treatments from the selected studies that share common proteomic targets.

## 3. Results

### 3.1. Data sources

We identified 27 studies focusing on ASD interventions (Figure 2), sourced from peer-reviewed journals. These studies were conducted across multiple continents, with the largest contributions from the United States (*n* = 12), followed by New Zealand (*n* = 2), Brazil (*n* = 2), Iran (*n* = 2), China (*n* = 2), and single studies from Jordan (*n* = 1), France (*n* = 1), Italy (*n* = 1), Chile (*n* = 1), Poland (*n* = 1), Slovakia (*n* = 1), and Vietnam (*n* = 1).

Our dataset included a mix of randomized controlled trials (*n* = 17), case reports (*n* = 2), *in vitro* studies (*n* = 1), and observational studies (*n* = 8). More detailed information about the study designs is available in Table S1.

### 3.2. Knowledge base

As shown in Table 1, the integrated analysis revealed over 8,000 potential drug candidates: 8,093 through therapeutic categories, 1,373 through proteomic targets, and 64 via pathways.

**Table 1:** Summary of potential drug candidates by therapeutic categories, proteomic targets, and biological pathways

Treatment	Compound	Therapeutic categories total	Proteomic targets total	Biological pathways total	Number of potential drugs through categories	Number of potential drugs through proteomic targets	Number of potential drugs through pathways
Methylphenidate	Methylphenidate	20	4	2	3,160	168	17
Memantine	Memantine	23	38	1	2,031	304	5
Atomoxetine	Atomoxetine	20	19	1	2,747	241	16
Fluoxetine+cyamemazine+valproic acid	Valproic acid	71	0	0	3,742	0	0
	Cyamemazine	11	4	0	3,062	157	0
	Fluoxetine	51	33	2	3,230	460	18
Fluoxetine+risperidone+loxapine	Loxapine	27	34	1	2,945	514	0
	Fluoxetine	51	33	2	3,230	460	18
	Risperidone	45	38	2	2,605	508	14
Fluoxetine+risperidone	Fluoxetine	51	33	2	3,230	460	18
	Risperidone	45	38	2	2,605	508	14
Fluoxetine+risperidone+melatonin	Melatonin	24	27	0	4,524	323	0
	Fluoxetine	51	33	2	3,230	460	18
	Risperidone	45	38	2	2,605	508	14
Controlled-release melatonin	Melatonin	24	27	0	4,524	323	0
N-acetylcysteine	Acetylcysteine	22	13	0	3,376	68	0
Buspirone	Buspirone	25	22	0	2,584	283	0
Intranasal oxytocin	Oxytocin	18	4	1	2,376	16	4
Vitamin D3	Vitamin D3	0	0	1	0	0	9
DHA; an omega-3 fatty acid	Doconexent	14	13	1	1,719	197	13
Vitamin D3+DHA	Vitamin D3	0	0	1	0	0	9
	Doconexent	14	13	1	1,719	197	13
Cannabidiol	Cannabidiol	59	66	0	2,456	565	0
Sulforaphane+risperidone	Sulforaphane	6	1	0	2,154	0	0
	Risperidone	45	38	2	2,605	508	14
Sulforaphane	Sulforaphane	6	1	0	2,154	0	0
Memantine+risperidone	Risperidone	45	38	2	2,605	508	14
	Memantine	23	38	1	2,031	304	5
Prednisolone	Prednisolone	45	7	2	3,053	93	18
Bumetanide	Bumetanide	30	24	1	3,087	112	8
Ketogenic diet+modified Atkins diet+low glycemic index treatments	Low glycemic index treatments	0	0	0	0	0	0
	Ketogenic diet	0	0	0	0	0	0
	Modified Atkins diet	0	0	0	0	0	0
Ubiquinol	Ubidecarenone	16	6	0	1,007	24	0
adrenal corticosteroid	Prednisolone	45	7	2	3,053	93	18
Luteolin	Luteolin	6	18	0	1,836	20	0
Umbilical cord blood infusion	Umbilical cord blood cells	0	0	0	0	0	0
Chondroitin sulfate+phosphatidylcholine+vitamin D3	Lecithin	13	1	0	1,273	0	0
	Vitamin D3	0	0	1	0	0	9
	Chondroitin sulfate	8	0	0	1,142	0	0
High protease pancreatic therapy	High-protease pancreatic enzyme	0	0	0	0	0	0
Single infusion of autologous umbilical cord blood	Autologous umbilical cord blood	0	0	0	0	0	0
Autologous bone marrow mononuclear cells	Autologous bone marrow mononuclear cells	0	0	0	0	0	0
Global	All				8,093	1,373	64

The resulting knowledge base consisted of 29 unique treatments, each corresponding to a distinct compound. Therapeutic categories were identified for 22 compounds, while seven (e.g., ketogenic diet, umbilical cord blood cells, and high-protease pancreatic enzyme) lacked categorization. Proteomic targets were available for 20 compounds; no targets were retrieved for nine, including valproic acid and Vitamin D3. Biological pathways remained undefined for six compounds due to limited data. Pathway information from SMPDB was available for 11 compounds (fluoxetine, risperidone, memantine, methylphenidate, atomoxetine, Vitamin D3, loxapine, oxytocin, bumetanide, prednisolone, and docosahexaenoic acid), allowing a comprehensive mapping of associated drugs.

The knowledge base also contained extensive, specific details for several compounds associated with numerous therapeutic categories, proteomic targets, and drug candidates for repurposing (Table 2).

**Table 2:** Selection of identified compounds/treatments and samples of associated drug candidates for repurposing

Compound/Treatment	Examples of candidates
Valproic acid	• Tacedinaline • Fimepinostat
Cannabidiol	• Theobromine • Naxifylline • Theophylline
Fluoxetine	• Tetrahydrobiopterin • Pyridoxal 5’- phosphate
Risperidone	• Carbamoylcholine • Fesoterodine
Prednisolone	• Paromomycin • Troxipide
Bumetanide	• Lornoxicam • Salsalate

### 3.3. Knowledge graphs

**Figure 3:**
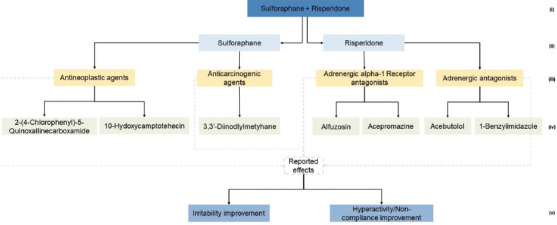
Therapeutic categories graph for sulforaphane + risperidone treatment. The numbers point to a given category level as follows: (i) Treatement; (ii) Compound; (iii) Therapeutic categories; (iv) Associated drug; (v) Observed effects.

**Figure 4:**
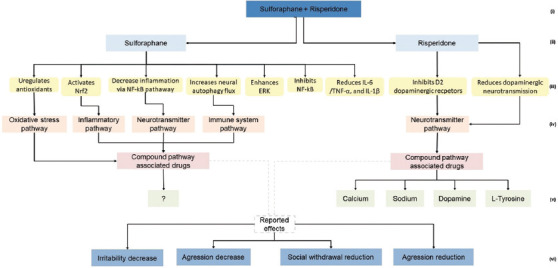
Biological pathways graph for sulforaphane + risperidone treatment. The numbers point to a given category level as follows: (i) Treatement; (ii) Compound; (iii) Pathway information; (iv) Compound global pathway; (v) Associated drugs; (vi) Observed effects. Abbreviations: ERK: Extracellular signal-regulated kinase; IL-1β: Interleukin 1 beta; IL-6: Interleukin 6; NF-κB: Nuclear factor kappa B; Nrf2: Nuclear factor erythroid 2-related factor 2; TNF-α: Tumor necrosis factor-alpha.

**Figure 5:**
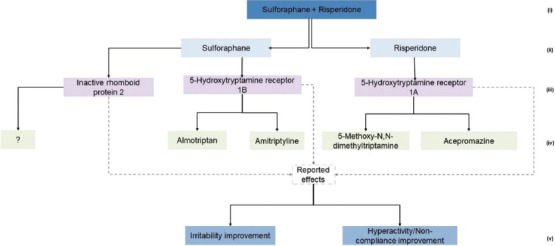
Proteomic targets graph for sulforaphane + risperidone treatment. The numbers point to a given category level as follows: (i) Treatement; (ii) Compound; (iii) Proteomic targets; (iv) Target-associated drugs; (v) Observed effects.

Using the available database information, several types of knowledge graphs were created at different levels of granularity. To ensure clarity and readability, the amount of information displayed on the graphs was intentionally limited. Comprehensive details are available in the interactive dashboard developed in this study. Examples of graphs related to the combined treatment of sulforaphane and risperidone for its “therapeutic categories” (Figure 3), “biological pathways” (Figure 4), and “proteomic targets” (Figure 5).

#### 3.3.1. Therapeutic categories graph

The therapeutic categories graph (Figure 3) starts by decomposing the combined treatment of sulforaphane and risperidone into its main components. Sulforaphane is linked to antineoplastic and anticarcinogenic agents. Risperidone is associated with adrenergic alpha-1 receptor antagonists and adrenergic antagonists. Each therapeutic category is linked to two additional drugs.

For example, under antineoplastic agents, 10-hydroxycamptothecin and 2-(4-chlorophenyl)-5-quinoxalinecarboxamide are listed. For adrenergic alpha-1 receptor antagonists, alfuzosin and acepromazine were mentioned. The therapeutic categories then converge into a “reported effects” box, showing improvements in irritability and hyperactivity/noncompliance. This graph suggested new candidates for ASD treatment, such as 10-hydroxycamptothecin and alfuzosin, which share therapeutic categories with existing ASD treatments.

#### 3.3.2. Biological pathways graph

Figure 4 illustrates an example of a biological pathways graph, which first links the components sulforaphane and risperidone to specific biological actions. Sulforaphane was associated with actions such as upregulating antioxidants, activating nuclear factor erythroid 2-related factor 2, inhibiting nuclear factor kappa B, enhancing extracellular signal-regulated kinase, increasing neuronal autophagy flux, and reducing interleukin 6/tumor necrosis factor alpha and interleukin 1 beta. These actions were connected to the predefined pathways, including oxidative stress, inflammation, neurotransmitter, and immune system pathways. Risperidone was linked to inhibiting D2 dopaminergic receptors and reducing dopaminergic neurotransmission. These actions were associated with the neurotransmitter pathway. The global biological pathways box combined these actions and pathways into a summarized view of the affected pathways. For risperidone, the graph included connections to drugs linked to the same SMPDB pathway. Where the SMPDB global compound pathway information for sulforaphane was missing, a question mark indicated the absence of data. The global pathways ultimately converged into a “reported effects” box, illustrating observed improvements in irritability and hyperactivity/non-compliance.

This graph identifies potential repositioning candidates such as calcium and carbon dioxide, which share biological pathways with risperidone but have not yet been tested for ASD.

#### 3.3.3. Proteomic targets graph

In Figure 5, the specific proteins targeted by sulforaphane and risperidone were identified. Sulforaphane targets included inactive rhomboid protein 2, while Risperidone targets 5-hydroxytryptamine receptors 1A and 1B. Each target was linked to additional drugs that interacted with the same proteins. For inactive rhomboid protein 2, a question mark indicated the absence of available data on drugs targeting this proteomic target, while for the 5-hydroxytryptamine receptors, linked drugs include 5-methoxy-N, N-dimethyltryptamine and acepromazine.

This graph suggests repositioning candidates like 5-methoxy-N, N-dimethyltryptamine, which targets proteins similar to risperidone but has not been tested for ASD. Additional examples of different graphs are available on the study’s dashboard.

### 3.4. Common proteomic targets

A detailed analysis of common proteomic targets among various treatments was conducted to assist in identifying treatments that share these targets. The findings for cannabidiol treatment, compared to the 28 other treatments, are summarized in Table 3.

**Table 3:** Comparison of shared proteomic targets between cannabidiol and other treatments

Main treatment	Comparator	Total common targets	Names of the targets
Cannabidiol	Methylphenidate	1	5-hydroxytryptamine receptor 1A
Cannabidiol	Memantine	8	Neuronal acetylcholine receptor subunit alpha-7; Cytochrome P450 2A6;Glycine receptor subunit alpha-1; Glycine receptor subunit beta; Cytochrome P450 2C19;Cytochrome P450 2B6;Glycine receptor subunit alpha-3; 5-hydroxytryptamine receptor 3A
Cannabidiol	Atomoxetine	2	Cytochrome P450 2D6; Cytochrome P450 2C19
Cannabidiol	Fluoxetine+cyamemazine+valproic acid	10	Cytochrome P450 2C9; ATP-dependent translocase ABCB1; Cytochrome P450 1A2; Cytochrome P450 3A4; 5-hydroxytryptamine receptor 2A; Cytochrome P450 3A5; Cytochrome P450 2D6; Cytochrome P450 2C19; Cytochrome P450 2B6; 5-hydroxytryptamine receptor 1A
Cannabidiol	Fluoxetine+risperidone+loxapine	11	Cytochrome P450 2C9; ATP-dependent translocase ABCB1; Cytochrome P450 1A2; Cytochrome P450 3A4; 5-hydroxytryptamine receptor 2A; Cytochrome P450 3A5; Cytochrome P450 2D6; Cytochrome P450 2C19; Cytochrome P450 2B6; 5-hydroxytryptamine receptor 3A; 5-hydroxytryptamine receptor 1A
Cannabidiol	Fluoxetine+risperidone	10	Cytochrome P450 2C9; ATP-dependent translocase ABCB1; Cytochrome P450 1A2; Cytochrome P450 3A4; 5-hydroxytryptamine receptor 2A; Cytochrome P450 3A5; Cytochrome P450 2D6; Cytochrome P450 2C19; Cytochrome P450 2B6; 5-hydroxytryptamine receptor 1A
Cannabidiol	Fluoxetine+risperidone+melatonin	13	Cytochrome P450 2C9; ATP-dependent translocase ABCB1; Indoleamine 2,3-dioxygenase 1; Cytochrome P450 1A2; Cytochrome P450 3A4; Cytochrome P450 1A1; 5-hydroxytryptamine receptor 2A; Cytochrome P450 3A5; Cytochrome P450 2D6; Cytochrome P450 2C19; Cytochrome P450 2B6; Cytochrome P450 1B1; 5-hydroxytryptamine receptor 1A
Cannabidiol	Controlled-release melatonin	7	Cytochrome P450 2C9; Indoleamine 2,3-dioxygenase 1; Cytochrome P450 1A2; Cytochrome P450 1A1; 5-hydroxytryptamine receptor 2A; Cytochrome P450 2C19; Cytochrome P450 1B1
Cannabidiol	Buspirone	8	ATP-dependent translocase ABCB1; Cytochrome P450 3A7; Cytochrome P450 3A4; 5-hydroxytryptamine receptor 2A; Cytochrome P450 3A5; Cytochrome P450 2D6; 5-hydroxytryptamine receptor 3A; 5-hydroxytryptamine receptor 1A
Cannabidiol	DHA (an omega-3 fatty acid)	4	Prostaglandin G/H synthase 1; Cytochrome P450 2C9; Peroxisome proliferator-activated receptor gamma; Prostaglandin G/H synthase 2
Cannabidiol	Vitamin D3+DHA (an omega-3 fatty acids)	4	Prostaglandin G/H synthase; Cytochrome P450 2C9; Peroxisome proliferator-activated receptor gamma; Prostaglandin G/H synthase 2
Cannabidiol	Sulforaphane+Risperidone	5	ATP-dependent translocase ABCB1; Cytochrome P450 3A4; 5-hydroxytryptamine receptor 2A; Cytochrome P450 2D6; 5-hydroxytryptamine receptor 1A
Cannabidiol	Memantine+Risperidone	13	ATP-dependent translocase ABCB1; Neuronal acetylcholine receptor subunit alpha-7; Cytochrome P450 3A4; 5-hydroxytryptamine receptor 2A; Glycine receptor subunit alpha-1; Cytochrome P450 2D6; Glycine receptor subunit beta; Cytochrome P450 2C19; Cytochrome P450 2B6; Cytochrome P450 2A6; Glycine receptor subunit alpha-3; 5-hydroxytryptamine receptor 3A; 5-hydroxytryptamine receptor 1A
Cannabidiol	Prednisolone	2	ATP-dependent translocase ABCB1; Cytochrome P450 3A4
Cannabidiol	Bumetanide	1	Prostaglandin G/H synthase 2
Cannabidiol	Ubiquinol	2	3-hydroxy-3-methylglutaryl-coenzyme A reductase; ATP-dependent translocase ABCB1
Cannabidiol	Adrenal corticosteroid	2	ATP-dependent translocase ABCB1; Cytochrome P450 3A4

Abbreviations: ATP: Adenosine triphosphate; DHA: Docosahexaenoic acid.

Cannabidiol and memantine shared eight common targets, including neuronal acetylcholine receptor subunit alpha-7 and cytochrome P450 2D6. Both treatments have shown improvements in social interaction. Cannabidiol was also involved in reducing anxiety and psychomotor agitation, while memantine was linked to improvements in communication.

Cannabidiol and the combination of fluoxetine, cyamemazine, and valproic acid shared 10 common targets, including cytochrome P450 2C9 and adenosine triphosphate-dependent translocase ABCB1. Both treatments reduced anxiety and improved social interaction. Cannabidiol was also associated with an increased number of daily meals and enhanced concentration, while the combination treatment demonstrated decreases in self-injurious behavior.

Cannabidiol and the combination of fluoxetine, risperidone, and loxapine shared 11 common targets, including cytochrome P450 2C9 and adenosine triphosphate-dependent translocase ABCB1. Both treatments improved social interaction and reduced aggressive behaviors. In addition, cannabidiol demonstrated reductions in anxiety and psychomotor agitation, while the combination treatment led to decreases in risperidone dosages.

Moreover, cannabidiol and the combination of fluoxetine, risperidone, and melatonin shared 13 common targets, including cytochrome P450 2C9 and adenosine triphosphate-dependent translocase ABCB1. Both treatments demonstrated improvements in social interaction and reductions in anxiety. Cannabidiol also showed an increased number of daily meals and enhanced concentration, while the combination treatment exhibited decreases in ADHD-like symptoms.

Cannabidiol, memantine, and risperidone shared 13 common targets, including neuronal acetylcholine receptor subunit alpha-7 and adenosine triphosphate-dependent translocase ABCB1. Both treatments showed reductions in irritability and stereotypic behavior. In addition, cannabidiol demonstrated improvements in social interaction, while the combination treatment was linked to reductions in hyperactivity.

## 4. Discussion

This study provides a comprehensive analysis of neuroinflammation’s role in ASD and explores the potential for drug repositioning in pediatric populations. By focusing on children aged 12 years or younger, we targeted a critical period of brain development, where interventions could have the most profound impact on long-term outcomes. This age group was chosen specifically because early childhood is a period of significant neurodevelopmental plasticity, during which therapeutic interventions might alter developmental trajectories more effectively than in older populations^21^. This approach, while narrowing the generalizability to older individuals, ensures that the findings are particularly relevant to the most vulnerable and developmentally significant phase of life.

Our results underscore the complexity of ASD and its associated neuroinflammatory pathways, reinforcing the multifaceted nature of the disorder. We identified more than 8,000 potential drug candidates through therapeutic categories, proteomic targets, and biological pathways, highlighting the vast potential for drug repositioning. This approach leverages existing drugs with known safety profiles, reducing the time and cost associated with developing new therapies. Notably, drugs such as cannabidiol, fluoxetine, and risperidone were mapped to multiple therapeutic categories and proteomic targets, indicating their broad potential utility in treating ASD symptoms in this population. For example, cannabidiol interacts with the adenosine receptor A1 and the serotonin receptor 5-HT1A, which may contribute to its anxiolytic and neuroprotective effects. Fluoxetine, a selective serotonin reuptake inhibitor, modulates serotonin levels, potentially improving mood and social behavior by enhancing synaptic plasticity. Risperidone, an atypical antipsychotic, antagonizes dopamine D2 and serotonin 5-HT2A receptors, reducing irritability and aggression in ASD. The common proteomic targets shared between these drugs, such as the serotonin receptor pathways, suggest potential synergistic effects that could stabilize neurotransmitter imbalances and modulate neuroinflammatory responses in ASD. Exploring these shared targets may lead to new combination therapies that maximize efficacy while minimizing side effects. Further clinical studies should investigate how these mechanisms interact in the context of neurodevelopment to refine and optimize treatment strategies for ASD. This aligns with the growing body of evidence supporting the role of neurotransmitter regulation and neuroinflammatory pathways in ASD.^22^ Another critical next step is the experimental validation of the identified drug candidates through *in vivo/in vitro* assays, as well as robust clinical trials.

One of the primary limitations of this study is the age range restriction, which, while deliberate, limits the applicability of the findings to older populations. This focus on early childhood, however, is justified by the critical nature of this developmental period, where the brain is most malleable and responsive to interventions.^23^ Future studies could expand the age range to assess whether the identified treatments maintain their efficacy in older children and adolescents, providing a more comprehensive understanding of the developmental trajectory of ASD and the long-term effects of these interventions.

Another limitation is the inherent bias introduced by excluding studies that did not report significant effects. While this exclusion criterion is necessary for identifying effective treatments, it potentially overlooks important data on less successful or neutral outcomes. Including these studies in future analyses could offer a more balanced view of the efficacy and limitations of various treatments, contributing to a more nuanced understanding of ASD therapies.

Furthermore, the selection of biological pathways in our study, while based on current scientific understanding, also presents a limitation. The pathways, such as inflammation, immune system, oxidative stress, neurotransmitter regulation, gene expression regulation, and the gut–brain axis, were chosen due to their frequent association with ASD in existing literature.^13^,^24^ However, the empirical validity of these pathways as central mechanisms in ASD remains an area of ongoing research and debate. While these pathways are supported by some studies, they may not fully encapsulate the complexity of ASD’s pathophysiology. Future research should validate these pathways through more extensive empirical studies to ensure they represent the most relevant biological processes in ASD.^25^

In addition, to provide a clear and concise visual representation of the results, we initially presented the graphs with a limited amount of information. This paper-based approach was intended to help readers quickly grasp the key findings without being overwhelmed by data. However, this limitation in the visualization of data could potentially obscure some valuable insights. To address this, we developed an interactive dashboard that allows users to explore the full range of data, including more detailed information on therapeutic categories, proteomic targets, and biological pathways. This tool helps overcome the limitations of static graphs by providing a more comprehensive and user-friendly exploration of the study’s findings.

The creation of an interactive dashboard to visualize and explore the extensive data collected in this study represents a significant advancement in making complex data more accessible and actionable for clinicians and researchers.^26^ This tool enables the dynamic exploration of therapeutic categories, biological pathways, and proteomic targets, facilitating the identification of potential drug candidates for further investigation. Future enhancements could include integrating more advanced bioinformatics tools and machine learning algorithms to predict treatment outcomes based on the data collected, thus personalizing treatment plans for individual patients.

Moreover, promising but poorly understood treatments, such as those involving cell-based therapies and specific dietary interventions, warrant further investigation. The lack of detailed data on the mechanisms of these treatments complicates the assessment of their efficacy. Future research should focus on elucidating these mechanisms, particularly in the context of neuroinflammatory processes and gut–brain interactions, which have shown potential in managing ASD symptoms.^27^

## 5. Conclusion

This study highlights the potential of drug repositioning as a promising strategy for addressing the complex neuroinflammatory pathways involved in ASD in children. By focusing on early childhood, a critical period for brain development, we have identified numerous potential therapeutic candidates that could be repurposed to target ASD’s multifaceted symptoms. For instance, cannabidiol, known for modulating serotonin and adenosine receptors, and fluoxetine, a selective serotonin reuptake inhibitor that enhances neuroplasticity, emerged as strong candidates for repurposing based on their interactions with key proteomic targets relevant to ASD. The results underscore the need for further research into the underlying mechanisms of cell-based therapy and dietary supplementation, particularly within the context of neuroinflammation and gut-brain interactions. Overall, this work lays a strong foundation for future studies aimed at developing more effective and personalized treatments for ASD, with the ultimate goal of improving the quality of life for children.

## Data Availability

A dashboard from this work is available through https://autism-research.univ-lille.fr/index.html.

## Supplementary File

**Table S1:** Study metrics

Authors, Year	Title	Country	Study type	Age range	Sample size	Male/Female	Diagnostic tools
Pearson *et al*.^1^	Effects of extended release methylphenidate treatment on ratings of ADHD and associated behavior in children with autism spectrum disorders and ADHD symptoms	USA	Within-subject; crossover; placebo-controlled clinical trial	7.1–12.7	24	19/5	ADI-R; ADOS; DSM-IV-TR; significant ADHD symptoms; clinical interviews; clinic observations; record reviews by licensed psychologists
Hardan *et al*.^2^	Efficacy and safety of memantine in children with autism spectrum disorder: Results from three phase 2 multicenter studies	USA	Randomized; double-blind; placebo-controlled; withdrawal trial; open-label extension trial	6–12	2123	1788/335	ADI-R; ADOS; DSM-IV-TR
Alsayouf *et al*.^3^	Atomoxetine treatment of ADHD symptoms in 3–6-year-old children with autism spectrum disorder: A retrospective cohort study	Jordan	Retrospective cohort study	3–6	133	114/19	DSM-5; CGI-S; direct observations of the patients’ behaviors; historical data; CARS2-ST; Vanderbilt ADHD diagnostic parent rating scale; clinic observations; parent reports
Luchelli and Bertschy^4^	Low-dose fluoxetine in four children with autistic spectrum disorder improves self-injurious behavior; ADHD-like symptoms; and irritability	France	Case report	6–12	4	3/1	DSM-5; ADI-R
Cortesi *et al*.^5^	Controlled-release melatonin; singly and combined with cognitive behavioral therapy; for persistent insomnia in children with autism spectrum disorders: a randomized placebo-controlled trial	Italia	Randomized; double-blind; placebo-controlled	4–10	160	132/28	ADI-R; ADOS-G; DSM-IV-TR; CBCL
Hardan *et al*.^6^	A randomized controlled pilot trial of oral N-acetylcysteine in children with autism	USA	Randomized; double-blind; placebo-controlled	3.2–10.7	33	31/2	ADI-R; ADOS; DSM-IV-TR; expert clinical evaluation; clinic visits; CGI-S
Chugani *et al*.^7^	Efficacy of low-dose buspirone for restricted and repetitive behavior in young children with autism spectrum disorder: A randomized trial	USA	Randomized; placebo-controlled; double-blind	2–6	166	137/29	ADI-R; ADOS; DSM-IV-TR
Parker *et al*.^8^	Intranasal oxytocin treatment for social deficits and biomarkers of response in children with autism	USA	Randomized; placebo-controlled; double-blind; parallel design	6–12	32	27/5	ADI-R; ADOS; DSM-IV-TR; DSM-5; telephone-screened; medical assessment; comprehensive psychiatric evaluation; clinic visits; Stanford-Binet Intelligence Scales fifth edition; CGI-S
Mazahery *et al*.^9^	A randomized-controlled trial of vitamin D and omega-3 long--chain polyunsaturated fatty acids in the treatment of core symptoms of autism spectrum disorder in children	New Zealand	Randomized; double-blind; placebo-controlled	2.5–8.0	117	100/17	DSM-5; standardized psychological tests; SRS; SPM
Silva *et al*.^10^	Evaluation of the efficacy and safety of cannabidiol-rich cannabis extract in children with autism spectrum disorder	Brazil	Randomized; double-blind; placebo-controlled	5–11	60	52/8	DSM-5; sociodemographic questionnaire; CARS; clinic observations; parent reports
	: Randomized; double-blind; and placebo-controlled clinical trial						
Momtazmanesh *et al*.^11^	Sulforaphane as an adjunctive treatment for irritability in children with autism spectrum disorder: A randomized; double-blind; placebo-controlled clinical trial	Iran	Randomized; double-blind; placebo-controlled	4–12	60	40/20	DSM-5; ADI-R; children’s behavioral examination; semi-structured interviews
Zimmerman *et al*.^12^	Randomized controlled trial of sulforaphane and metabolite discovery in children with autism spectrum disorder	USA	Randomized; placebo-controlled; double-blind	3–12	45	40/5	ADOS; VABS-II; semi-structured interview; Leiter International Test of Intelligence-Revised
Mazahery *et al*.^13^	A randomized controlled trial of vitamin D and omega-3 long chain polyunsaturated fatty acids in the treatment of irritability and hyperactivity among children with autism spectrum disorder	New Zealand	Randomized double-blind; placebo-controlled	2.5–8.0	73	60/13	DSM-5
Ghaleiha *et al*.^14^	Memantine as adjunctive treatment to risperidone in children with autistic disorder: a randomized; double-blind; placebo-controlled trial	Iran	Randomized; double-blind; placebo-controlled	4–12	40	23/17	DSM IV-TR; ADI-R; behavioral observation; semi-structured interview
Wink *et al*.^15^	A randomized placebo-controlled pilot study of N-acetylcysteine in youth with autism spectrum disorder	USA	Randomized; double-blind; placebo-controlled	4–12	31	24/7	DSM-IV; ADI-R; clinical interview; Leiter International Test of Intelligence-Revised; CGI-S
Fieiras *et al*.^16^	Risperidone and aripiprazole for autism spectrum disorder in children: an overview of systematic reviews	Chile	Overview of systematic reviews (SRS)	0–12	NA	NA	DSM-5; ADI-R; ADOS; CARS; ASIEP-2; CSBS (Caregiver Questionnaire); SCATA; ToPP; CBS; NCBRS; clinic observations; parent reports
Brito *et al*.^17^	Effect of prednisolone on language function in children with autistic spectrum disorder: a randomized clinical trial	Brazil	Randomized; double-blind; placebo-controlled	3–7	40	40/0	DSM-IV; CARS; clinic observations; parent reports
Dai *et al*.^18^	Improved symptoms following bumetanide treatment in children aged 3−6 years with autism spectrum disorder: A randomized; double-blind; placebo-controlled trial	China	Randomized; double-blind; placebo-controlled; parallel-group	3–6	119	100/19	ADI-R; ADOS; DSM-5; SRS; CARS; clinic observations; parent reports
Zhang *et al*.^19^	Symptom improvement in children with autism spectrum disorder following bumetanide administration is associated with decreased GABA/glutamate ratios	China	Randomized; placebo controlled; double-blind	3–6	83	65/18	ADI-R; ADOS; DSM-5; CARS; clinic observations; parent reports
Zarnowska *et al*.^20^	Therapeutic use of carbohydrate-restricted diets in an autistic child; a case report of clinical and 18FDG PET findings	Poland	Case report	6	1	1/0	DSM-IV-TR; psychological and psychiatric evaluations; CARS; WISC-R; clinic observations; parent reports
Gvozdjáková *et al*.^21^	Ubiquinol Improves Symptoms in Children with Autism	Slovakia	Open trial	3–6	24	17/7	DSM IV; CARS; clinic observations; parent reports
Duffy *et al*.^22^	Corticosteroid therapy in regressive autism: a retrospective study of effects on the FMAER; language; and behavior	USA	Retrospective study	3–5	44	36/8	DSM-IV; diagnostic by academic child neurologists and/or psychiatrists, and/or psychologists
Asadi and Theoharides^23^	Corticotropin-releasing hormone and extracellular mitochondria augment immunoglobulin E-stimulated human mast-cell vascular endothelial growth factor release; which is inhibited by luteolin	USA	*In vitro* study	NA	NA	NA	NA
Simhal *et al*.^24^	Changes in the geometry and robustness of diffusion tensor imaging networks: Secondary analysis from a randomized controlled trial of young autistic children receiving an umbilical cord blood infusion	USA	Randomized; placebo-control; double-blind; single site; prospective	2–7	165	131/34	ADI-R; ADOS; DSM-5; CGI-S
Antonucci *et al*.^25^	Clinical experience of integrative autism treatment with a novel type of immunotherapy	Italy	Open-label; non-controlled; retrospective analysis	3.1–4.5	3	3/0	ATEC; clinical diagnostic
Pearson *et al*.^26^	Pancreatic replacement therapy for maladaptive behaviors in preschool children with autism spectrum disorder	USA	Randomized; placebo-controlled; double-blind parallel group; delayed-start	3–6	190	150/40	SCQ; DSM-IV-TR; ADI-R
Carpenter *et al*.^27^	White matter tract changes associated with clinical improvement in an open-label trial assessing autologous umbilical cord blood for treatment of young children with autism	USA	Phase I open-label trial	2–6	25	17/2	DSM-5; ADOS; ADI-R; CGI-S
Thanh *et al*.^28^	Outcomes of bone marrow mononuclear cell transplantation combined with interventional education for autism spectrum disorder	Vietnam	Open-label uncontrolled clinical trial	3.0–7.4	30	25/5	DSM-5; CARS

Abbreviations: ADHD: Attention-deficit/hyperactivity disorder; ADI-R: Autism diagnostic interview-revised; ADOS-2: Autism diagnostic observation schedule, second edition; DSM-IV-TR: Diagnostic and statistical manual of mental disorders, 4^th^ edition, text revision; DSM-5: Diagnostic and statistical manual of mental disorders, 5^th^ ed.ition; CGI-S: Clinical global impressions-severity; CARS: Childhood autism rating scale; CBCL: Child behavior checklist; VABS-II: Vineland adaptive behavior scales, second edition; SRS: Social responsiveness scale; SPM: Sensory processing measure; WISC-R: Wechsler intelligence scale for children-revised; SCQ: Social communication questionnaire; ATEC: Autism treatment evaluation checklist; FMAER: Frequency modulated auditory evoked response; ASIEP-2: Autism screening instrument for educational planning, second edition; CSBS: Communication and symbolic behavior scales; SCATA: Social Communication assessment for toddlers with autism; ToPP: Test of pretend play; CBS: Child behavior scale; NCBRS: Nisonger child behavior rating scales; 18FDG PET: 18 F-fluorodeoxyglucose positron emission tomography; GABA: Gamma-aminobutyric acid

**References**

1. Pearson DA, Santos CW, Aman MG, *et al*. Effects of extended release methylphenidate treatment on ratings of attention-deficit/hyperactivity disorder (ADHD) and associated behavior in children with autism spectrum disorders and ADHD symptoms. *J* *Child Adolesc Psychopharmacol*. 2013;23(5):337-351. doi:10.1089/cap.2012.0096
2. Hardan AY, Hendren RL, Aman MG, *et al*. Efficacy and safety of memantine in children with autism spectrum disorder:Results from three phase 2 multicenter studies. *Autism.* 2019;23(8):2096-2111. doi:10.1177/1362361318824103
3. Alsayouf HA, Alsarhan O, Khreisat W, Daoud A. Atomoxetine treatment of attention deficit/hyperactivity disorder symptoms in 3-6-year-old children with autism spectrum disorder:A retrospective cohort study. *Children (Basel)*. 2024;11(2):163. doi:10.3390/children11020163
4. Lucchelli JP, Bertschy G. Low-dose fluoxetine in four children with autistic spectrum disorder improves self-injurious behavior, ADHD-Like symptoms, and irritability. *Case Rep Psychiatry*. 2018;2018:6278501. doi:10.1155/2018/6278501.
5. Cortesi F, Giannotti F, Sebastiani T, Panunzi S, Valente D. Controlled-release melatonin, singly and combined with cognitive behavioural therapy, for persistent insomnia in children with autism spectrum disorders:A randomized placebo-controlled trial. *J* *Sleep Res*. 2012;21(6):700-709. doi:10.1111/j.1365-2869.2012.01021.x.
6. Hardan AY, Fung LK, Libove RA, *et al*. A randomized controlled pilot trial of oral N-acetylcysteine in children with autism. *Biol Psychiatry*. 2012;71(11):956-961. doi:10.1016/j.biopsych.2012.01.014
7. Chugani DC, Chugani HT, Wiznitzer M, *et al.* Efficacy of low-dose buspirone for restricted and repetitive behavior in young children with autism spectrum disorder:A randomized trial. *J* *Pediatr*. 2016;170:45-53.e1-4. doi:10.1016/j.jpeds.2015.11.033
8. Parker KJ, Oztan O, Libove RA, *et al*. Intranasal oxytocin treatment for social deficits and biomarkers of response in children with autism. *Proc Natl Acad Sci U S A*. 2017;114(30):8119-8124. doi:10.1073/pnas.1705521114
9. Mazahery H, Conlon CA, Beck KL, *et al*. A randomised-controlled trial of vitamin D and omega-3 long chain polyunsaturated fatty acids in the treatment of core symptoms of autism spectrum disorder in children. *J* *Autism Dev Disord*. 2019;49(5):1778-1794. doi:10.1007/s10803-018-3860-y.
10. Silva EAD Jr., Medeiros WMB, Santos JPMD, *et al*. Evaluation of the efficacy and safety of cannabidiol-rich cannabis extract in children with autism spectrum disorder:Randomized, double-blind, and placebo-controlled clinical trial. *Trends Psychiatry Psychother*. 2024;46:e20210396. doi:10.47626/2237-6089-2021-0396.
11. Momtazmanesh S, Amirimoghaddam-Yazdi Z, Moghaddam HS, Mohammadi MR, Akhondzadeh S. Sulforaphane as an adjunctive treatment for irritability in children with autism spectrum disorder:A randomized, double-blind, placebo-controlled clinical trial. *Psychiatry Clin Neurosci*. 2020;74(7):398-405. doi:10.1111/pcn.13016.
12. Zimmerman AW, Singh K, Connors SL, *et al*. Randomized controlled trial of sulforaphane and metabolite discovery in children with autism spectrum disorder. *Mol Autism*. 2021;12(1):38. doi:10.1186/s13229-021-00447-5
13. Mazahery H, Conlon CA, Beck KL, *et al*. A randomised controlled trial of vitamin D and omega-3 long chain polyunsaturated fatty acids in the treatment of irritability and hyperactivity among children with autism spectrum disorder. *J* *Steroid Biochem Mol Biol*. 2019;187:9-16. doi:10.1016/j.jsbmb.2018.10.017
14. Ghaleiha A, Asadabadi M, Mohammadi MR, *et al*. Memantine as adjunctive treatment to risperidone in children with autistic disorder:A randomized, double-blind, placebo-controlled trial. *Int J Neuropsychopharmacol*. 2013;16(4):783-789. doi:10.1017/S1461145712000880
15. Wink LK, Adams R, Wang Z, *et al*. A randomized placebo-controlled pilot study of N-acetylcysteine in youth with autism spectrum disorder. *Mol Autism*. 2016;7:26. doi:10.1186/s13229-016-0088-6
16. Fieiras C, Chen MH, Escobar Liquitay CM, *et al*. Risperidone and aripiprazole for autism spectrum disorder in children:An overview of systematic reviews. *BMJ Evid Based Med*. 2023;28(1):7-14. doi:10.1136/bmjebm-2021-111804
17. Brito AR, Vairo GPT, Dias APBH, Olej B, Nascimento OJM, Vasconcelos MM. Effect of prednisolone on language function in children with autistic spectrum disorder:A randomized clinical trial. *J* *Pediatr (Rio J)*. 2021;97(1):22-29. doi:10.1016/j.jped.2019.10.012
18. Dai Y, Zhang L, Yu J, *et al*. Improved symptoms following bumetanide treatment in children aged 3-6 years with autism spectrum disorder:A randomized, double-blind, placebo-controlled trial. *Sci Bull (Beijing)*. 2021;66(15):1591-1598. doi:10.1016/j.scib.2021.01.008
19. Zhang L, Huang CC, Dai Y, *et al*. Symptom improvement in children with autism spectrum disorder following bumetanide administration is associated with decreased GABA/glutamate ratios. *Transl Psychiatry*. 2020;10(1):9. doi:10.1038/s41398-020-0692-2
20. Żarnowska I, Chrapko B, Gwizda G, NocuńA, Mitosek-Szewczyk K, Gasior M. Therapeutic use of carbohydrate-restricted diets in an autistic child;A case report of clinical and 18FDG PET findings. *Metab Brain Dis*. 2018;33(4):1187-1192. doi:10.1007/s11011-018-0219-1
21. GvozdjákováA, KucharskáJ, OstatníkováD, BabinskáK, Nakládal D, Crane FL. Ubiquinol improves symptoms in children with autism. *Oxid Med Cell Longev*. 2014;2014:798957. doi:10.1155/2014/798957
22. Duffy FH, Shankardass A, McAnulty GB, *et al*. Corticosteroid therapy in regressive autism:A retrospective study of effects on the frequency modulated auditory evoked response (FMAER), language, and behavior. *BMC Neurol*. 2014;14:70. doi:10.1186/1471-2377-14-70
23. Asadi S, Theoharides TC. Corticotropin-releasing hormone and extracellular mitochondria augment IgE-stimulated human mast-cell vascular endothelial growth factor release, which is inhibited by luteolin. *J* *Neuroinflammation*. 2012;9:85. doi:10.1186/1742-2094-9-85
24. Simhal AK, Carpenter KLH, Kurtzberg J, *et al*. Changes in the geometry and robustness of diffusion tensor imaging networks:Secondary analysis from a randomized controlled trial of young autistic children receiving an umbilical cord blood infusion. *Front Psychiatry*. 2022;13:1026279. doi:10.3389/fpsyt.2022.1026279
25. Antonucci N, Pacini S, Ruggiero M. Clinical experience of integrative autism treatment with a novel type of immunotherapy. *Madridge J Vaccines*. 2019;3(1):71-76. doi:10.18689/mjv-1000116
26. Pearson DA, Hendren RL, Heil MF, McIntyre WR, Raines SR. Pancreatic replacement therapy for maladaptive behaviors in preschool children with autism spectrum disorder. *JAMA Netw Open*. 2023;6(11):e2344136. doi:10.1001/jamanetworkopen.2023.44136
27. Carpenter KLH, Major S, Tallman C, *et al*. White matter tract changes associated with clinical improvement in an open-label trial assessing autologous umbilical cord blood for treatment of young children with autism. *Stem Cells Transl Med*. 2019;8(2):138-147. doi:10.1002/sctm.18-0251
28. Nguyen Thanh L, Nguyen HP, Ngo MD, *et al*. Outcomes of bone marrow mononuclear cell transplantation combined with interventional education for autism spectrum disorder. *Stem Cells Transl Med*. 2021;10(1):14-26. doi:10.1002/sctm.20-0102
